# Nature Cure and Non-Communicable Diseases: Ecological Therapy as Health Care in India

**DOI:** 10.3390/ijerph14121525

**Published:** 2017-12-07

**Authors:** Joseph S. Alter, R. M. Nair, Rukmani Nair

**Affiliations:** 1 Department of Anthropology, University of Pittsburgh, Pittsburgh, PA 15217, USA; 2 Bapu Nature Cure Hospital and Yogashram, Gandhi Samarak Nidhi, Mayur Vihar Phase-1, New Delhi 110091, India; drrmnair@bnchy.org (R.M.N.); rashmi@bnchy.org (R.N.)

**Keywords:** India, Nature Cure, noncommunicable diseases, holistic health, alternative medicine, bioecology

## Abstract

With rapidly increasing rates of non-communicable diseases, India is experiencing a dramatic public health crisis that is closely linked to changing lifestyles and the growth of the middle-class. In this essay we discuss how the practice of Nature Cure provides a way of understanding the scale and scope of the crisis, as it is embodied, and a way to understand key elements of a solution to problems that the crisis presents for institutionalized health care. As institutionalized in contemporary India, Nature Cure involves treatment and managed care using earth, air, sunlight, and water as well as a strict dietary regimen. In this regard, the essay shows how Nature Cure’s bio-ecological orientation toward public health, which is grounded in the history of its modern incorporation into India, provides an expansionist, ecological model for holistic care that counters the reductionist logic of bio-medical pharmaceuticalization.

## 1. Introduction

### Overview and Background

India is in the midst of a major public health crisis as a result of rapidly increasing rates of cardiovascular heart disease, diabetes, osteoarthritis, chronic obstructive pulmonary disease (COPD) and other diseases closely linked to life-style in general and unhealthy patterns of consumption in particular. Changes in life-style and patterns of consumption are unambiguously linked to dramatic changes in the country’s political economy and the rapid growth and prosperity of the middle-class. In this essay we show how the practice of Nature Cure, a system of medicine rooted in Gandhian principles that is certified by the Indian government, provides a model for the care of patients at risk for the development of Non-Communicable Diseases (NCDs), for the prevention of increased risk of complications associated with these diseases, and a reduction of cost in the long-term care and management of aging patients. Nature Cure in contemporary India, which derives directly from late 19th and early 20th century forms of European practice, involves treatment using earth, air, sunlight, water and a regulated, primarily raw vegetarian diet to treat a spectrum of medical conditions. It is now often referred to a Naturopathy, a designation which reflects early 20th century professionalization. However, many practitioners in India continue to use the term Nature Cure, or Indian language equivalents such as *Prakrtic Chikitsa*. We use Bapu Nature Cure Hospital and Yogashram as an example of an effective person-centered, local response to a problem of national and international health. In contrast to the reductionist logic of biomedicine, we refer to this as an “expantionist” approach to medicalized public health. In doing so, we hope to integrate analytical and applied perspectives to build on recent work in medical anthropology that critically examines the professionalization of alternative medicine in contexts of medical pluralism and in relation to questions of holism [1,2,3,4,5,6].

By “expansionism” we mean a form of personalized public health that is the antithesis of medical reductionism, as reductionism constitutes a broad, epistemic orientation toward biomedical health care. Reductionism is a powerful tool that guides medical research and practice in terms of deterministic logic. The power of this operational logic in producing effective forms of treatment is unambiguous in both clinical and public health contexts. However, determinism is also what has created problems of alienation at different levels of human experience, especially those experiences that are social, ecological and personal. As manifest in Nature Cure generally, and as embodied in the practice of Bapu Nature Cure Hospital and Yogashram specifically, we show how an “expansionist” orientation toward therapy reflects a shift in focus toward expansive ecological holism and away from both isolated pathology and reactive, defensive prevention.

Our focus here is on holism as a way of conceptualizing public health, broadly defined. While generalized notions of preventative wellness and health education have become common in many contexts, expansionism, as manifest clearly in Nature Cure, is characteristic of forms of treatment that are radical and pointedly reactionary, albeit often in subtle ways [7]. Just as reductionism presumes that efficacy at the level of the individual is what is ultimately important and of absolute value, expansionism involves a more dialectical logic in which a person’s health is subsumed into a synthesis that is inherently bio-ecological [8]. To be sure, expansionism has problems and limitations, but we are interested in understanding how holism is embodied and the operational structure of community health based on the embodied materialism of ecological principles. In other words, we focus on the manipulation of bodies in the environment as a means of producing an improved sense of health and wellbeing rather than on ideals and the disembodied idealism of healing as an end unto itself.

## 2. Nature Cure and the Crisis of Public Health in Contemporary India

### 2.1. Nature Cure: Practice and Theory

Not only is Nature Cure unique among the plurality of medical systems in India, it is based on a theory of health and disease that is fundamentally ecological. Although generalized “natural” wellness is a feature of “alternative” healing which has gained currency in various circles worldwide, Nature Cure should not be confused with early 20th century “New Age” spirituality or its various derivative forms of practice. There are some interesting and important points of historical overlap in the development of each, especially as concerns Theosophy in the history of Indian modernity. But, contrary to what one might expect—given Swami Vivekanand’s influence in the late 19th century—and, despite the impact of Orientalized yoga on embodied spirituality more generally [9,10], Nature Cure in India—including, perhaps most surprisingly, the practice of asana and pranayama—reflects a distinctly modernized articulation of early 20th century radical theory and practice as articulated by German secular pragmatists who were less mystically inspired than mechanically inclined to bring nature into the urban world of the new middle-class [11]. This techno-holism makes Nature Cure more relevant to 21st century public health issues in India than is the case with other medical systems [8,12].

In most fundamental terms, Nature Cure is based on the principle that the body heals itself and that therapy facilitates a process of organic recovery. Therapy involves steam baths, spray showers, hot and cold-water compresses, enemas, wet cloth wraps, mud baths, sun baths, breathing exercises, walking, and various dietary regimens incorporating raw or minimally cooked vegetables, whole grains, fruits, and nuts. What are called “diseases” in biomedical terms are, in essence, both symptoms of compromised health and positive signs of recovery in process. They are not entities unto themselves, regardless of the fact that the direct cause of a disease may be a virus, a parasite, a cancerous tumor or insulin deficiency. Symptoms that are used to establish biomedical diagnosis, and test results that reveal pathology, are, from the perspective of Nature Cure, positive signs that the body is trying to heal itself, and that it needs help. Help is provided by removing “toxic” elements that inhibit recovery, not by combating disease. Thus, in conjunction with a regimen of enemas, followed by a diet of fruit juice and sprouted whole grains, fasting can be understood as an “antidote” to an extended pattern of consumption that is the primary cause of diarrhea and high fever, as both the fever and diarrhea are natural, physiological processes of organic adjustment rather than simply pathological signs of infection. This fundamentally blurs the line between prevention and cure, which, quite apart from the question of Nature Cure’s practical value as triage in the context of a, say, a viral epidemic, does have important implications for the extended treatment of life-style “diseases” that involve complex links between the body, ecology and the environment.

Nature Cure provides a distinct understanding of physiology and pathology that reflects an broad, expansionist, environmental perspective on health rather than a reductive biological perspective on prevention and cure, keeping in mind that integrative medicine in contemporary practice bridges the divide between holism and reductionism in important ways [13,14].

As intimated above, from the perspective of Nature Cure, a body is both an environment unto itself, as well as an organism that reflects health in relation to larger, ecological processes. Thus, the body has independent, organic agency as a function of its position within an ecological matrix. The way in which a body is said to “heal itself” reflects an understanding of this kind of agency rather than a contrived anthropomorphized—and moralized—form of cultural agency shrouded in the entelechy of biological purpose. To be sure, Nature Cure is based on ideological principles of bio-morality, but in terms of a critique of modernity and the consequences of triumphal cultural production, rather than in terms of an ontology of intrinsic value. This finds expression in a utopian vision of ecological equipoise. Biomedicine, by contrast, is a directional project that imposes the will of culture on nature with the utopian prospect of liberating the body and society from the burden of disease. Both visions have their place in contemporary India. But equipoise may have particular relevance in relation to the ongoing epidemiological transition and a national crisis of health that is ecological by nature.

### 2.2. Non-Communicable Diseases: The Indian Crisis in Global Perspective

As in many other countries, life-style diseases now cause more deaths in India than do infectious diseases such as malaria, tuberculosis, and cholera [15]. Fifty-three percent of all deaths are now correlated with Non-Communicable Diseases (NCDs), including cardiovascular diseases, 24%, COPD, 11%, cancer, 6%, diabetes, 2%. A range of other chronic diseases make up the remaining 10% [16]. Having declined significantly over the course of the past 50 years, 36% of all deaths are still correlated with communicable diseases, maternal and prenatal health problems, and nutritional deficiency [17]. Although it stands out on several measures, India is by no means unique. A 2011 conference organized under the auspices of the WHO produced some striking statistics on NCDs in the context of international and global health [18]. What is striking is that the steady decrease in infectious diseases reflects a profound shift in the burden of disease within a relatively compressed period of time. Between 2005 and 2015 alone the WHO projected that there would be a 15% reduction in deaths caused by nutritional deficiency, maternal and infant health problems and communicable diseases [19]. This is obviously good. But it is also indicative of a serious public health problem, namely the complex morbidity of those who live longer. One approach would be to conceptualize the problem holistically and develop solutions that build on this conceptualization.

For a number of reasons that reflect genetic predisposition, rapid rates of urbanization, a surge in the consumption of fat, salt and sugar, and dramatic changes in life expectancy due to the control of infectious diseases, India has one of the world’s highest rates of cardiovascular heart disease among men and women under the age of sixty [20,21]. There has also been a dramatic increase in the incidences of diabetes [22,23].

NCDs are a global problem with specific public health and economic implications for lower income countries [24]. Globally 63% percent of all deaths are caused by NCDs, of which 25% or 9.1 million are premature and avoidable. Ninety percent of premature deaths from NCDs occur in low and lower middle-income countries, with heart disease and stroke being the leading cause. Based on projections, the number of premature deaths from heart disease will increase from 6.1 to 8.2 million in low income countries, and from 7.3 to 9 million in lower middle-income countries. In upper middle income countries the death rate is projected to remain steady at 2.6 million. In developing countries, the top 10 risk factors correlated with premature deaths are all for NCDs. 6.2 million deaths can be attributed to high blood pressure, which leads all other factors including tobacco use by over 2.3 million. By comparison, 2.7 million deaths can be attributed to high blood glucose which constitutes the third greatest risk factor for premature death. This is followed by physical inactivity, to which 2.6 million deaths can be attributed [25,26,27,28,29,30].

Given that many NCDs are progressive diseases that produce increased morbidity over time, especially during the most productive years of adult life, there is a direct correlation between broad patterns of exposure to risk and a cycle of household poverty that further increases risk and exacerbates the health impact of all of the major NCDs, especially cardiovascular disease and diabetes.

Broad demographic changes in population aging and urbanization, correlated with a political economy of consumption and inactivity, can lead directly to a loss of household income as a result of unhealthy behaviors. When risk behaviors involving food, inactivity, tobacco use and alcohol consumption cause NCDs, the economic impact on households is multidimensional. First, income is lost as a consequence of not being able to work. Expenses for extended treatment are often very high—if adequate treatment is available at all—and the limited availability of treatment often leads to a further reduction in physical health. This can produce a heavy drain on limited household resources as expensive treatment is extended over a number of years while a patient’s health deteriorates.

Based on WHO estimates, India lost 9 billion dollars in national income as a result of premature deaths caused by heart disease, stroke and diabetes in 2005. The projected loss of income through 2015 is 237 billion dollars. Remarkably, 80% of these deaths could easily be prevented by means of diet reform, increased physical activity and a significant reduction in the consumption of tobacco. A reduction in the death rate by just 2% annually over ten years would produce a 15-billion-dollar economic gain in national income. Clearly NCDs are a significant drag on overall economic growth and development [19]. Given this larger, economic impact, as well as the multidimensional structure of risk—involving the food industry, private and public medical services and a spectrum of life style issues that have a bearing on physical activity—the WHO has pointed out that NCDs present a major political challenge and problem for development and are not just a problem of public health.

Tobacco is, of course, an iconic example of how health, politics and the global economy are intertwined. Another example, with different but equally important dynamics, is the pharmaceutical industry, encompassing biomedicine and, increasingly, other medical systems such as *ayurveda*, *unani* and *siddha*, as well as the wellness industry of herbal tonics and supplements [31].

However, food itself is also intimately linked to health in often subtle and complex but also direct and discernable ways [32]. Deep and extensive chains of production, processing, marketing and distribution generate a phenomenal array of packaged goods, ranging from basic, branded staple commodities to highly refined specialty “health food” items constituted of artificial ingredients that remove the risk of sugars, fats and salt. Commodity chains that link point of production to point of sale to point of consumption are rightly targeted by governments for regulation, but market dynamics and a porous global economy often works against these regulations, as in the case of tobacco. In terms of food production and processing, a simple intervention involves the replacement of trans fats with polyunsaturated fats. But the real, operational problem is at the level of enforcement and labeling, as these are only really effective when supported by a structure of economic incentives and risk awareness education.

From a broad evolutionary perspective on holism, the problem is not only whether a particular item of food is unhealthy, but what volume of mass produced food—which is inherently unhealthy—is being consumed. In other words, one could argue that the correlation between NCDs and diet is a problem of total volume of processed food being consumed rather than a problem of the specific nutritional configuration and composition of the food item in question. Trans fats and sodium are dangerous in relatively small amounts, but they are at one end of a spectrum that includes food items high in saturated fats and cholesterol, foods that carry a high glycemic load and refined foods that have a high proportion of starch. At the other end of the spectrum are fruits and vegetables. In the calculus of over consumption relative to metabolism, obesity is simply a sign of maladaptation to an environment that has become completely domesticated over the course of the past 10,000 years. As such, generalized obesity and overweight can be understood as an encompassing risk factor for the development of many NCDs, especially as people live longer; but it can also be interpreted as symptomatic of a larger, ecological problem in the relationship that humans have contrived with the environment. Weight reduction, which involves exercise and diet modification, is a crude but useful measure of incremental health improvement, and serves to reinforce a fundamental principle in the logic of diet reform—moderation in all things.

In most general terms, the NCD health crisis in India is a function of the time compression of an epidemiological transition that has condensed the cumulative effect of changes in diet, life expectancy and life-style into the collective experience of one or two generations [33]. In the discussion which follows we show that institutionalized Nature Cure provides a comprehensive framework for understanding the scale and scope of the crisis in contemporary India, as it is embodied, and that it is within this framework that radical forms of treatment using earth, air, sunlight, water and dietetics constitute a holistic form of medical intervention and management.

### 2.3. Nature Cure in the Context of Indian Health Care

Public health and medicine in India is based on the principles, programs and procedures of biomedical science. However, the Indian government has also institutionalized a policy of medical pluralism, providing support, certification and legal status for a range of different medical options, including *ayurveda*, yoga, *unani*, *siddha*, naturopathy and homeopathy, among others [34]. Until recently the administration of these different systems was centralized under the auspices of the Ministry of Health and Family Welfare. Since 2014, all non-biomedical systems of health care are organized within the Ministry of AYUSH (Ayurveda, Yoga and Naturopathy, Unani, Siddha and Homeopathy) [35]. Politics, education and economics are important to consider in understanding the history of India’s policy on AYUSH pluralism [36,37,38]. Similarly, the changing landscape of global health has shaped and will continue to reshape various systems of medicine in their local articulation. Thus, health tourism is having a powerful impact on *ayurvedic* practice, especially in Kerala and Uttarakhand [39,40]. Religious nationalism has contributed to the institutionalization of distinctions between *ayurveda*, *unani* and *siddha*, despite histories of continuity and shared knowledge [41,42]. In terms of globalization, the practice of yoga has become iconic of the relationship among spirituality, self-discipline, health and physical fitness [43,44].

In slightly different ways, AYUSH systems of medicine have become highly commercialized and commoditized. Yoga is a multi-billion-dollar service industry, with niche markets in clothing and tourism [45,46]. The industrialization of *ayurveda*, *unani*, *siddha* and homeopathy is clearly manifest in the scale and scope of international pharmaceutical companies such as Dabur, Himalaya and Hamdard. To some extent the commercialization of AYUSH systems reflects consumer demand for alternatives to biomedicine, although “demand” is probably generated as much by marketing as anything else [31]. Pharmaceutical companies profit as a consequence of the fact that distress is symptomatic of human biology. Any and all forms of medicine are only provisionally and contingently successful in saving lives and eliminating pain. People want more from medicine than can be provided by medicine, particularly as it tends to be professionalized and institutionalized. Nevertheless, in their complex, fully medicalized plurality, AYUSH systems highlight the way in which public health concerns are not directly congruent with reductive, pharmaceutical interventions, or with the practice of remedial medicine as such.

In light of this, the political history and cultural significance of Nature Cure is somewhat unique and especially relevant to a consideration of chronic, life-style diseases. In a number of important ways it is different from all other AYUSH medical systems, even though it shares a degree of common cultural history with homeopathy [12].

First, Nature Cure took shape as a form of reactionary alternative medicine in south-central Europe during the peak of the industrial revolution and developed into a critique of standard biomedicine around the turn of the 19th century at roughly the same time as germ theory was being applied in practice, and as vaccination protocols became standardized. Nature Cure was and continues to be opposed to fundamental aspects of biomedical theory and practice, including key elements of etiology, diagnosis and treatment, as well as basic ontological assumptions about the nature of the body, health and pathology. In the context of colonial India, the reactionary nature of Nature Cure provided a way to develop a sense of self-care that worked against the ever-increasing conjunction of imperialism, medicine and public health.

Second, Nature Cure’s popularity in India is to a large extent based on advocacy by Mohandas K. Gandhi who believed that hydrotherapy and diet reform in particular were integral features of the larger struggle to gain independence from colonial rule [36]. Although many people find it difficult to fully appreciate, Gandhi believed, fundamentally, that the violence in medicine was as serious a problem as communal violence, racial prejudice and colonial oppression in society at large. As reflected in his program of action, Nature Cure and diet reform provided the means by which the body could take on moral and ethical significance in community development and constructive work. As such, the health of the body was programmatically connected to the health and welfare of the community and the nation as a whole, and this connection was intimate, direct and grounded in action rather than idealistically figurative or purely symbolic of abstract correlations.

Third, unlike other AYUSH systems, (with the exception of homeopathy) Nature Cure does not reflect—or carry the cultural burden—of tradition and regional heritage. Knowledge about it is not rooted in a body of literature with strong religious overtones. It is not associated with religious history and, despite its point of origin in Europe, it is not linked to colonial forms of power [11,36]. Indeed, Nature Cure’s reactionary and critical perspective on medical practice is countercultural rather than normative, but also active and contemporary rather than anachronistic. To be sure, its development was inspired by ideas and practices associated with sacred springs and mineral baths in medieval Europe. However, from the very beginning of its formal conception Nature Cure has continued to evolve into a self-consciously modern form of practice fully engaged with technology and mechanized forms of industrial modernity. It is not based on the aesthetic principles of picturesque romanticism, or on a mystical sense of rustic empathy with pristine, wild nature.

Fourth, Nature Cure’s modernity is manifest in a distinctly “scientific” and rational conceptualization of nature in relation to the environment and the body. Although all AYUSH systems of medicine have come to be defined in terms of a discourse of contemporary laboratory science, based on experimentation of various kinds—and a reductive logic of cause and effect—Nature Cure is defined in terms of what might be called a late 18th century conception of natural philosophy involving observation and direct experience. In other words, it reflects an analytical perspective on nature and natural processes that emerges from within the history and philosophy of modern science, as science developed and changed at a critical juncture in time—the late 19th century. Nature Cure manifests a trajectory of reasoning about health that is highly critical of biomedical determinism, but from the vantage point of a distinct tangential development in the logic of precisely the same science that gave rise to the power of determinism and reductionist reasoning.

Fifth, although Nature Cure took institutionalized form in the context of central Europe in the 19th century, it reflects the materialization of a history of concerns about health that parallels the global dynamics of industrialization and, in particular, the emergence of middle-class distinctions within the political economy of urban growth and development [8]. Thus, Nature Cure may be understood in terms of the embodiment of opposition to alienating features of work and labor during the course of a key demographic transition from rural agricultural production to capital accumulation and wage labor. As one dimension of this, it also reflects a critical reaction against the industrialized, mass production of food, perhaps most clearly articulated in Sylvester Graham’s advocacy for whole wheat alternatives to bleached and chemically processed flour in 19th century New England, and the consolidation of advocacy against meat eating as expressed in the principles of the International Vegetarian Union of Germany in the early 20th century. The specific social class dynamics of this broad movement are undoubtedly complex and multi-dimensional, involving fine grained distinctions of displaced self-doubt, self-reflexive critiques of conspicuous consumption, as well as a kind of middle-class Rousseauian romanticism that took shape as bourgeois nostalgia for rural life in the countryside, but without the critical insight of historical memory for what peasant life was really like. In any case, the cultural history of Nature Cure is intimately linked to a social history of class distinctions on a global scale.

In sum, to understand the “culture” of Nature Cure, in South Asia and elsewhere, it is important to recognize the extent to which this culture has taken shape over the course of at least a century based on the exchange of ideas through networks of likeminded individuals in the United States, England, Europe, and parts of Asia [43]. These individuals—on the “margins of the fringe”, even when in positions of significant wealth and power—were likeminded as a result of their critical perspective on the public health consequences of modernity. They also held similar views on the relatively new culture of mass consumption that characterized the global middle-class at the turn of the century. Thus, one of the most important things that distinguishes Nature Cure from other AYUSH systems of medicine is the fact that its popularity is much more closely tied to questions of public health and social class in contexts of dynamic transnationalism than to cultural tradition, ethnic heritage and nationalism within a narrow framework of colonialism [11].

## 3. Results: Bapu Nature Cure Hospital and Yogashram

Bapu Nature Cure Hospital and Yogashram (BNCHY) provides a useful case study for understanding how Nature Cure is institutionalized, what treatment is like, and, most particularly, what kind of patients, with what configuration of symptoms, make use of Nature Cure facilities [37]. Overall this provides critical insight on health management strategies that have a bearing on important cultural, economic and medical considerations [47].

BNCHY is a twenty bed hospital that was established in 1984 under the auspices of Nature Cure and Yoga Trust, a non-profit NGO. In line with Gandhian principles, the hospital was established at Gandhi Nidhi at Patparganj on the east bank of the Yamuna River, across from Raj Ghat and the Gandhi Memorial Museum. The hospital was founded just thirty years ago, in what was then a village market center on the agricultural fringe of the capital. Its institutional history is linked to the establishment of the Akhil Bharatiya Prakritic Chiktisa Parishad (ABPCP) in 1946, and to the work of C.A. Menon on behalf of this organization. In conjunction with his interest in Nature Cure, Menon was a Gandhian and became active in Vinoba Bhave’s *bhoodan* (land donation) movement in the early 1950s. He sought to embody his commitment to social justice, economic reform and political activism, in the tradition of other early Gandhians, in particular Manibhai Desai and Balkova Bhave, both of whom were instrumental in the early development of Nisargopachar, an experimental Nature Cure center established in 1946 by Gandhi in the village of Urulikanchan near Pune. Menon’s management of the ABPCP in Delhi provided a national framework for the regional work being done in Urulikanchan, and the BNCHY may be seen as a project that grew out of his focus on Nature Cure promotion, professional training, and publishing.

As an All India Federation established by Gandhi, the ABPCP highlights the way in which Nature Cure can reflect principles of non-violence, simplicity and moderation. An unassuming and self-consciously anachronistic organization it has been supplanted—both ideologically and institutionally—by the Central Council for Research on Yoga and Naturopathy (CCRYN). In many ways the BNCHY clearly articulates modern goals of scientific practice and medical pluralism as manifest in the CCRYN, under the auspices of AYUSH. However it also continues to promote professionalized Nature Cure along lines set down by Gandhi, Bhave and Menon in the 1940s. In this respect it is representative of almost all other Nature Cure clinics and hospitals in India, the primary difference being its unique historical credentials and its institutional partnership with the Central Government Health Scheme.

Since 1984 BNCHY has expanded and developed and is currently in the process of adding a unit with 100 additional beds along with new equipment, necessary additional infrastructure and support facilities. However, basic treatment remains essentially the same. This involves a range of different kinds of hydropathic techniques including spinal spray baths, full emersion baths, fomentation baths, whirlpool baths, arm and leg baths and both steam and dry sauna baths as well as enemas. To facilitate various forms of hydrotherapy there are a number of different kinds of tubs, in particular one designed for *kati snan*, a type of seated hip bath integral to Gandhian practice. There is a section for mud baths as well as for sun baths and electric chromo-therapy. Massage is an integral aspect of therapy and BNCHY has several rooms dedicated to this form of treatment as well as more contemporary forms of physiotherapy. As the name itself would suggest, yoga is an integral feature of modern Nature Cure and the Hospital has a designated area where patients are guided through a regimen of *asana* and *pranayama* every morning.

Treatment by the medical staff at BNCHY is provided for specific medical problems, as discussed in more detail below. However, in keeping with its broad, Gandhian orientation, the organization is also deeply invested in the promotion of Nature Cure as a health maintenance life-style based on several kinds of community outreach and education. This, in conjunction with the structure of care provided for patients—both resident and non-resident—produces an effective framework for the control and management of a large number of chronic life-style diseases. What the BNCHY does very effectively is what might be called proactive embodied health education.

Health education is only effective if knowledge is turned into practice, and if the benefits of practice are confirmed by experience and reinforced in various ways. In many ways, turning knowledge into practice is an uphill battle, the challenge being to work against complacency, the comfort of passivity and indiscriminate excessive consumption. It is with reference to this point that Nature Cure presents a unique, “dialectical” intervention in health education. By dialectical we mean, simply, the way in which two related but somewhat contradictory principles—holistic “prevention” and reductive “cure”—are synthetically reconciled in expansionist practice. Each will be examined in turn.

In part because of Gandhi’s influence, Menon’s efforts and the support structure of the centralized Gandhian Trust, the BNCHY has established a strong connection to public service institutions through its structured health scheme called the Central Government Health Scheme, CGHS. As a recognized and certified hospital, the BNCHY provides treatment for government employees at a set rate paid directly by the employer or employing agency to the hospital. While the vast majority of dispensaries and clinics in the CGHS system provide biomedical treatment, twelve hospitals in different parts of the country are empaneled for the provision of *ayurvedic* treatment, two for *unani* and two for naturopathy. The maximum rates that can be charged for specific kinds of treatment are set by the government. Thus a “four-week package of nine naturopathic treatments including special diet” is set at Rs 8000 (USD 134.00). A whole-body mud bath costs Rs 150 (USD 2.30) and a thermoleum bath, using colored light, costs Rs 50 (USD 0.75) [48]. As described below, BNCHY patients, many of whom are Central Government employees, or employees in one of over thirty government corporations and companies such as Housing and Urban Development, Power Finance and the Food Corporation of India, are treated for specific medical conditions.

Drawing on its structural position within the CGHS, BNCHY has developed a program of wellness workshops for the employees of the numerous government bureaucracies that it serves as well as the employees of large public and private corporations and both state and municipal agencies and institutions, such as the Delhi Police, Bharat Petroleum, and the Delhi Development Authority. These workshops are conducted free of charge, and are designed to promote proactive healthcare based on exercise, stress reduction and management and diet modification, as well as to provide general information about Nature Cure treatment for chronic NCDs. The workshops are participatory and engaging. Short lectures are followed by group activities, in particular guided instructions on yoga *asana* (postural exercise) and *pranayama* (breathing exercise) as well as practical demonstrations on how to select and prepare healthy food.

While there are no data upon which to make an assessment of the effectiveness of these workshops, the simple math of the savings in prevention relative to the cost of future treatment is unambiguously clear. To be sure, the BNCHY stands to benefit financially from the CGHS arrangement, and there is an element of marketing and self-promotion in the corporate workshops. But, as a legacy of Nehruvian democratic socialism, the CGHS makes it possible for Nature Cure to institutionalize a radical program of ecological health based on the principle that symptoms are a sign of healing, and that community health can be achieved by working with the elements of the environment rather than against the manifestations of disease, even when they are embodied. In this sense the BNCHY workshops reflect the principles of public health activism within the rubric of Nehruvian socialism, recognizing the problems and limitations of this with respect to class formations and interests.

From the standpoint of a skeptical practitioner of biomedicine, focused on the evidentiary criteria of science to measure efficacy and to make assessments of value, Nature Cure as “treatment” for heart diseases and diabetes can be discounted as quackery on account of interventions using water, earth and air. Nevertheless, it is important to point out that many of the life-style changes in diet and exercise that are recommended within the framework of Nature Cure are very similar to those recommended by practitioners of integrative medicine, many of whom approach the problem of management and treatment of NCDs from a perspective that places emphasis on evidence based interventions [49,50,51,52,53]. Heart disease and diabetes, among most other NCDs, are progressive, multi-dimensional conditions that become more serious and more expensive to treat over time. An important consideration, therefore, is the question of whether or not patients integrate life-style lessons from workshops into their everyday lives. Although no data on this key factor are available, it is useful to think of the risk factors and symptoms of NCDs—both early and late stage—as moving targets that can be managed effectively in relation to medical criteria that change significantly during the course of a person’s life. If the trajectory of this change leads only toward more narrowly defined, invasive, and pharmaceutically structured interventions, a person is progressively “reduced down” to the constellation of diseases he or she is diagnosed with and the overwhelmingly negative picture of health that is associated with the implementation of this reductionist logic.

## 4. Analysis and Examples: Expansionist Perspectives on Ecological Health

In many ways Nature Cure turns the problem of impersonal medicalization on its head. Patients subscribe to a disciplinary regimen based on ideals of collective self-control and the principle of *swaraj* (self-rule in the Gandhian sense) as an antidote to alienated, isolated subjection. In this regard the regimen of treatment at BNCHY is very Gandhian, even though it has been thoroughly monetized under the rubric of the CGHS. Whatever the implications of this may be with respect to empowerment, pastoral governmentality and questions of autonomy in everyday life, it is a regimen of treatment for individuals that highlights problems of “public” health, expanding outward from the individual to society and the environment rather than projecting solutions inward to the body and its pathology. And this is its fundamental value, ideological rhetoric, imperfect implementation and problems of compliance notwithstanding.

A small sample of forty-seven patient files drawn from the 2012 medical records of the BNCHY provides a perspective on how the philosophy of Nature Cure works in practice. The sample, which is approximately 10% of the number of patients per year, is broadly representative of cases treated at BNCHY. Although arbitrarily selected by an office manager at the request of the authors, the sample does not reflect a random selection of cases. Pseudonyms are used to ensure anonymity.

Twenty-six patients in the sample are men and twenty-one women, ranging in age from twenty-four to eighty-eight with the median being sixty-seven. Half of all patients are between sixty and seventy years old. While a few patients are obese, it is somewhat surprising that only half of the patients are overweight, and then only slightly. Since almost all patients diagnosed with hypertension (HTN) are on management drug regimens to reduce blood pressure, only six patients registered stage-two hypertension. All are part of the CGHS, each one going through a regimen of inpatient care for approximately one month.

What is striking about the sample is that each patient suffers from a number of different health problems and has suffered from these for many years. Each person identified a configuration of problems, ranging from two to nine. Five is the average with the most common number of complaints being four as reflected in the cases of twelve patients. A majority of all patients had been suffering from one or more of these health problems for at least two years. Many patients in their late sixties and early seventies had been suffering for upward of five years, many for ten years or more, and a significant number for more than twenty years.

Based on biomedical diagnostic categories used by the BNCHY, over fifty different conditions are identified in the sample. However, almost all complaints are associated with a smaller set of ‘diseases’, in particular insulin dependent and non-insulin dependent diabetes, hypertension, spondylosis and osteoarthritis. Many patients with diabetes have common symptoms of thirst, hunger and excessive urination.

At the time of admission, patients meet with a doctor who provides a provisional diagnosis on the basis of self-reporting and a review of the patient’s medical record. Patients then meet with a doctor every day to review their treatment.

After their initial meeting with the doctor patients identify their “complaints”. This often duplicates information collected during the admission interview. However, the identification of complaints is more subjective, often combining the use of biomedical terminology with more idiosyncratic feelings, and, significantly, almost always includes a temporal picture of development. Following on this, the doctor elicits a more comprehensive, but cryptic “history of the illness” as well as the patient’s account of his or her more general medical history, family medical history, and gynecological and obstetric history if relevant. Personal health habits are also recorded.

Following on the collection of “vital data,” including weight and height, blood pressure, and heart rate, the doctor performs a general exam of each patient, recording characteristics of build, appearance, cyanosis, dystonia, pallor, icterus, clubbing and jaundice, a characteristic that is deemed to be different from icterus. The general exam is followed by a “systemic examination” of cardio-vascular, respiratory, renal, abdominal, neurological locomotor, skin, eyes, and also an obstetric and gynecological examination.

The process by which information and data is collected from a person shifts back and forth from the particular to the general and from objective to subjective, always involving an extended dialogue between doctor and patient. Significantly, all of this is operationalized into the framework of an encompassing “provisional diagnosis” which includes three protocols (1) facial diagnosis; (2) iridiagnosis; and (3) clinical diagnosis. Facial diagnosis is a protocol developed in Germany by Louis Kuhne in the late 19th century. It entails an interpretation of the “signs” of poor health as manifest in facial features and physiologic asymmetry. Iridiagnosis is a protocol that involves a diagnostic interpretation of the color configuration of the iris. Clinical diagnosis, in this context, involves standard biomedical tests that are indicated, such as blood sugar tests and ECG (electrocardiogram). The provisional diagnosis is then synthesized into a final diagnosis, but treatment is always prescribed on the basis of the more holistic picture manifest in the encompassing multi-dimensional, interactive diagnostic procedure as a whole. In conjunction with this, patients are reexamined on a daily basis during the course of treatment, both with regard to vital statistics as well as more subjective criteria.

Although there are very common patterns, and specific techniques are standardized on the basis of objectified methods and procedures, a very distinctive and important feature of Nature Cure at BNCHY is the way in which treatment is personalized. Each person’s regimen, beginning in the early morning and running through each evening, serves to locate that person in a highly structured socio-ecological environment wherein that person is at once active and passive, unique and typical in ways that are markedly different from common experiences in everyday life outside the facility. Although often mechanical, and always structured and regimented, treatment is stimulating, visceral and sensory in uncommon ways. This produces a distinct sense of embodiment in relation to treatment as a whole and is an integral feature of the way in which bio-ecological sensibility emerges as a component of healing. In essence specific features of embodied experience—including the measurement and recording of vital data, the visceral experience of bowel evacuation, the ineffable torpidity of massage and the feeling of being covered with wet earth and of washing it off when it dries—establish a holistic matrix of ecological health by connecting a person’s body to the environment.

The underlying social production of this matrix deserves particular emphasis, if only because the sensory aspects of embodiment tend to localize and particularize a set of actions that involve doctors, technicians, therapists, attendants and support personnel who cook, clean and maintain facilities, gardens and lawns. In other words, it is important to conceptualize BNCHY as a place where the social production of health is comprehensively structured with reference to very specific features of pathology on the one hand and equally specific features of ecology on the other.

Overall this produces what we are calling an expansionist articulation of experiential public health as distinct from the articulation of reductionist medicalization based either on prophylactic prevention or treatments for symptomatic pathology and disease. Many if not all patients at BNCHY continue to take drugs prescribed by biomedical physicians, but these regimens are incorporated into the purview of Nature Cure’s more holistic, expansionist parameters concerning the body, health and the environment. Several cases described in what follows will provide examples of treatment in relation to diagnosis.

Mrs. Sheila Singh is sixty years old, weighs 130 kg, and has a blood pressure reading of 140/90. She was admitted in mid-August 2012 with complaints of “loose motion with gas and acidity, for the past ten years; insulin-dependent diabetes with polyuria and polyphagia, for the past thirty years; hypertension with occipital headache and swelling in both legs, for the past thirty years; bronchial asthma with breathlessness, for the past twenty-five years; hypothyroidism, for the past twenty-five years, and a fatty liver.” She spent 28 days at the facility, leaving in mid-September.

Although her stay was relatively long, there is no way in which the treatment she received during this time can be understood as a “cure.” To be sure, Mrs. Singh lost three kilograms, had a reduced BP reading of 130/80 upon discharge, and felt much better. With blood sugar levels under control she was not constantly thirsty and did not constantly feel the need to urinate. Flatulence, acidity, loose motions and chest congestion had been reduced. Lumber pain, which had developed mid-way through the treatment, was gone by the end of her stay.

There is nothing dramatic in Mrs. Singh’s case, and one could argue that it would be better to see more tangible results of efficacy—greater weight loss, for example, and a further reduction in blood pressure. However, if we view the process of her treatment as part of an end unto itself rather than an intervention leading to a definitive, somewhat abstract “end”—some approximation of Mrs. Singh’s health as it might have been if she had not become dependent on insulin thirty years ago—than we can better understand how the treatment makes her feel in relation to the social and environmental ecology of BNCHY as the nexus of an expansionist ecosystem of health.

Mrs. Singh’s regimen begins every morning at six am with yoga *asana*, *pranayama* and meditation. The routine is conducted on the roof top of the hospital, under a thatched roof with adobe walls reminiscent of an idealized, rustic village home. It lasts for an hour and is done in the company of other patients, each with their own set of complaints, under the direction of a certified yoga therapist. Specific, personalized Instructions enable Mrs. Singh to avoid or modify techniques for postures that are contraindicated for her specific set of problems. In keeping with various forms of modern *asana* and *pranayama*, yoga at BNCHY is done either vigorously or sedately, depending on preferences and/or prescriptions, and Mrs. Singh is able to pace herself accordingly, just as the therapist is able to adapt instructions to accommodate different levels of skill and fitness. At the end of the routine Mrs. Singh is served herbal tea.

The remainder of the day is structured around prescribed meals and two 1.5 to 2-h long therapy sessions, one in the morning and one in the late afternoon.

Following yoga, Mrs. Singh had a glass of aloe vera juice every day for five days, followed by lime juice in water for two days, and then seasonal fruit juice every day for the remainder of her stay. On the first day she had par-boiled sprouted grains in the morning at approximately 8:30, followed by vegetable *idli* (steamed rice flour dumplings) for two days, vegetable *daliya* (wheat porridge) for one day, vegetable *upma* (pancake) for one day, and then mixed seasonal fruits for the next six days. For the remainder of her stay, morning meals consisted variably of one or the other of these items, or else *khichardi* (lentil and rich stew) and raw vegetable salad. Her mid-day meal, taken at about 1:30, was more standardized, usually consisting of three whole-wheat *chapatis* (unleavened bread), a serving of boiled vegetables, *dal* (lentil soup), salad, and seasonal fruit. The *dal* and vegetables were unspiced, very lightly salted and prepared without oil. At five in the evening Mrs. Singh had a tumbler full of vegetable soup every day. Her evening meal, taken at approximately 8 pm, was also standardized, including either a serving of *daliya* or two *chapatis* with boiled vegetables, salad, and a glass of milk.

Mrs. Singh’s therapy sessions were quite variable from day to day, each session being structured with reference to her general diagnosis, but also modified daily based on changes in constitution and overall health. A morning session might include either a full body massage or a partial massage to a particular area—back, lower back, legs, lower legs—followed by mud packs to the eyes, forehead and abdomen, a liver mudpack, and a hot chest pack. In the afternoon she would almost always have a head bath, alternating hot and cold compresses to the abdomen, and “direct mud” to the abdomen and legs. Occasionally she would have a hot or cold knee bath and a spinal bath.

Mr. Shankar is seventy-two ears old, weighs 75 kg, and has a medicated BP reading of 122/80. When admitted in late January he had “lower back pain, for the past three years; knee pain with stiffness and swelling, for three years; high blood pressure, for twenty years; and increased blood sugar, for twenty years.” Diagnosed with diabetes, he is not dependent on insulin. Following 29 days of treatment, Mr. Shankar was discharged with his “blood sugar and blood pressure under control.” He was advised to continue with his newly established dietary regimen and to “take a hip bath, make use of a liver pack and a hot compress as needed to relieve discomfort in the affected areas, and for a better life.”

The structure and schedule of Mr. Shankar’s regimen is identical to Mrs. Singh’s, although quite different in the details. In the morning he has various combinations of a full body massage, mud packs to the legs, knees and lower back, mud packs to the eyes and abdomen, hot, cold and normal hip baths, and enemas. In the late afternoon he has a combination of treatments including hot compresses to the abdomen, hot foot bath with salt, a liver mud pack, and knee fomentation.

Mr. Shankar’s diet is broadly the same as Mrs. Singh’s, but also slightly different. His morning meal is more variable than hers from day to day, but across the same range of items, adding mint *chattni* to the portions of *upma* and *idli*. For the mid-day meal he has two rather than three *chapatis* with boiled vegetables and *dal*. In the evening he had seasonal fruit juice every day for the duration, following six days of purifying cumin water at the beginning of his stay. His evening meal is always the same, including two whole-wheat *chapatis* boiled vegetables, *dal*, salad and milk.

Mr. Datta is seventy-nine years old, weighs 78 kg, and has a medicated BP reading of 120/76. He was admitted in mid-April with “irritable bowel syndrome (IBS), coronary artery disease, knee pain and diabetes.” He had angioplasty with a stent one year before undergoing Nature Cure treatment. When discharged in mid-May, Mr. Datta “had major relief from IBS, lowered blood sugar levels and a significant reduction in the pain and stiffness in his knees.” He was advised to continue with the prescribed diet, his specific regimen of yogic exercises, and to regularly drink plenty of water.

Mr. Datta’s regimen of treatment is similar in structure, but also quite different from Mr. Shankar’s and Mrs. Singh’s. It includes a full body massage every day for twenty-nine days; a steam bath with a cold chest pack on seven different days; daily combined applications of a local mud pack to the abdomen and eyes; daily tepid hip baths and daily liver mud packs; two days of special, extra one-hour long yoga sessions; twelve treatments of foot and arm baths on alternating days; twenty-two consecutive days of alternating hot/cold compresses applied to the lower back and legs; and twelve applications of a chest and abdomen cool wet flannel cloth pack.

Mr. Datta’s diet is subtly but significantly different from both Mrs. Singh’s and Mr. Shankar’s, including only *khichardi* for the mid-day meal, soup in the late afternoon and papaya in the evening along with three whole-wheat *chapatis*, boiled vegetables, *dal* and salad. *Khichardi* and papaya are specifically indicated for the treatment of IBS.

Mrs. Bhagwat is sixty-five years old, weighs 120 kg and has a BP reading of 140/90. When admitted in early October 2013, she had a pain in her left knee with crepitus and stiffness. She also had dilated veins in both legs, for the past four years, hypertension with occipital headache and nausea, for the past year, hypothyroidism, for the three months, and a pain in the left side of her abdomen for the past month. After thirty-three days of treatment her weight came down to 119 kg and there was a reduction in the intensity of her leg and knee pain as well as a contraction in the dilation of her varicose veins. She was advised to “continue eating a bland diet and to practice yoga.”

Mrs. Bhagwat’s treatment involved twenty-five physiotherapy sessions; thirty applications of eye and abdomen mud packs; seventeen special additional yoga sessions; thirty applications of a hot/cold alternating compress to her knees, legs and abdomen; thirty hot foot and arm baths; four applications of liver packs; two ice massage sessions; and sixty-eight wet flannel mustard powder cloth poultice packs to places on her legs and knees. These are especially indicated for varicose veins.

Mrs. Bhagwat’s morning meal includes the same range and variation of items as for other patients—vegetable *idli*, vegetable *daliya*, and vegetable *upma*. At the beginning of her stay both mid-day and evening meals consisted of three *chapatis*, boiled vegetables, *dal*, and salad. After eleven days her evening meal was changed to *khichardi*. After twenty-two days the mid-day meal was also changed to *khichardi.* Thus, her diet shifted gradually from whole wheat to whole-grain unpolished rice.

In most general terms, all patients at BNCHY are prescribed a combination of treatments with water (hydrotherapy), earth (mud baths), sun (heliotherapy) and air (pranayama breathing exercise), in conjunction with prescribed regimens of exercise (walking and/or yogic postural exercises) and diet. Variation within this range of interventions is determined by diagnosis, an evaluation of medical history of chronic problems and an assessment of immediate risk factors associated with symptoms such as hypertension, asthma, and arthritis. For example, certain postural exercises that strain joints are not recommended for patients with severe arthritis and/or loss of bone density, and specific dietary modifications are prescribed depending on blood sugar testing and BMI assessment.

## 5. Conclusions

Viewed in terms of individualized treatment, or even from the perspective of reductive prevention, skeptics could argue that Nature Cure is not particularly effective. Nevertheless, some patients do get significantly better, and the BNCHY has helped a large number of patients recover. They leave feeling physically much better as well as having a better sense of the relationship among treatment, health and life-style. But to focus on these cases, exceptional or not, is beside the point we are making here, even though they demonstrate the professionalism and skill of the medical staff.

Nature Cure is concerned with treatment that promotes organic healing as a process that changes the nature of the environment a person inhabits. Treatment expands gradually into the ecology of techno-material holism that defines this embodied space, the shift in Mrs. Bhagwat’s dietary regimen being simply one case in point of a process that is incremental and engrossing. The process is dynamic, sometimes subtle—as in Mrs. Bhagwat’s case, Mr. Shankar’s cumin water and Mrs. Singh’s regimen of aloe vera juice—pointedly tautological, but, ultimately, endless. But it is endless in a non-pejorative sense. Therapy is intended to establish an embodied way of life that encompasses the symptoms of disease rather than be a means by which to overcome diseases in order to regain health that has somehow been lost. If you have suffered for years and years from a chronic condition this is profoundly reassuring, and can be empowering.

In the context of India’s contemporary health crisis, relatively simple interventions could dramatically reduce risk among the increasingly expanding, affluent middle-class. These interventions boil down to not smoking or drinking alcohol to excess, eating healthier food and getting more exercise. To the extent that yoga is exercise, Nature Cure provides a comprehensive approach to prevention. However valuable this may be, and however effective the BNCHY workshops are in promoting this kind of holistic health, prevention for the sake of prevention is, again, somewhat beside the point we are making here. From the perspective of the BNCHY, prevention works holistically if it is proactive as an ecology of health that engages the middle-class from within the parameters of a toxic modernity that has been thoroughly embodied for several generations. Mrs. Singh, who has been sick for over thirty years, helps to define the parameters of this ecology in relation to her body.

In keeping with the basic principles of Nature Cure’s radical theory of health, BNCHY therapy is holistically ecological, and is focused on the ways in which this ecology can be expanded and tailored to accommodate the organic healing processes manifest in the protracted symptoms embodied by patients such as Mrs. Singh, Mrs. Bhagwat, Mr. Shankar and Mr. Datta. In a very important way, and in a way that reflects Gandhian principles, the BNCHY provides personalized holistic treatment for patients so that the community they constitute defines an alternative ecology that serves as an expansive antidote to a crisis of individualized middle-class public health.

## References

[1] Baer H.A., Beale C., Canaway R., Connolly G. (2012). A dialogue between naturopathy and Critical Medical Anthropology: What constitutes holistic health?. Med. Anthropol. Q..

[2] Baer H.A. (2012). Rejoinder: A long and convoluted journey: Medical pluralism, naturopathy, and Critical Medical Anthropology. Med. Anthropol. Q..

[3] Baer H.A. (2004). Toward an Integrative Medicine: Merging Alternative Therapies with Biomedicine.

[4] Broom A., Doron A., Tovey P. (2009). The inequalities of medical pluralism: Hierarchies of health, the politics of tradition and the economies of care in Indian oncology. Soc. Sci. Med..

[5] Nissen N., Manderson L. (2013). Researching alternative and complementary therapies: Mapping the Field—Introduction. Med. Anthropol..

[6] Tippens K.M., Oberg E., Bradley R. (2012). A dialogue between naturopathy and Critical Medical Anthropology: Toward a broadened conception of holistic health. Med. Anthropol. Q..

[7] Alter J.S., Sharma C.S. (2016). Nature cure treatment in the context of India’s epidemiological transition. J. Integr. Med..

[8] Alter J.S., Singer M. (2016). Medicine, alternative medicine and political ecologies of the body. A Companion to Environmental Health: Anthropological Perspectives.

[9] De Michelis E. (2004). A History of Modern Yoga: Patañjali and Eastern Esotericism.

[10] Singleton M., Goldberg E. (2014). Gurus of Modern Yoga.

[11] Alter J.S. (2014). Nature cure and ayurveda: Nationalism, viscerality, and bio-ecology in India. Body Soc..

[12] Jansen E. (2016). Naturopathy in South India: Clinics between Professionalization and Empowerment.

[13] Aggarwal M., Aggarwal B., Rao J. (2017). Integrative medicine for cardiovascular disease and prevention. Med. Clin. N. Am..

[14] Mehta D. (2017). Integrative medicine and cardiovascular disorders. Prim. Care.

[15] WHO Major NCDs and Their Risk Factors. http://www.who.int/ncds/en/.

[16] WHO NCD Country Profiles—India. http://www.who.int/countries/ind/en/.

[17] WHO Noncommunicable Diseases Now Biggest Killers. http://www.who.int/mediacentre/news/releases/2008/pr14/en/.

[18] WHO Combating NCDS: Protecting Health, Promoting Development. http://www.who.int/nmh/events/2011/ncds_booklet_2011.pdf.

[19] WHO Facing the Facts: The Impact of Chronic Disease in India, n.d.. http://www.who.int/chp/chronic_disease_report/media/INDIA.pdf.

[20] Pandit-Agrawal D., Khadilkar A., Chiplonkar S., Khadilkar V., Patwardhan V. (2017). Screening score for early detection of cardio-metabolic risk in Indian adults. Int. J. Public Health.

[21] Singh A., Dixit S. (2016). Lower socio-economic status and cardiovascular disease: Role of healthcare facility and policy in India. Indian J. Community Health.

[22] Anjana R.M., Deepa M., Pradeepa R., Mahanta J., Narain K., Das H.K., Adhikari P., Rao P.V., Saboo B., Kumar A. (2017). Prevalence of diabetes and prediabetes in 15 states of India: Results from the ICMR-INDIAB population-based cross-sectional study. Lancet Diabetes Endocrinol..

[23] Gwatidzo S.D., Williams J.S. (2017). Diabetes mellitus medication use and catastrophic healthcare expenditure among adults aged 50+years in China and India: Results from the WHO study on global ageing and adult health. BMC Geriatr..

[24] Tabutin D., Masquelier B. (2017). Mortality Inequalities and Trends in Low- and Middle-Income Countries, 1990–2015. Population.

[25] Arokiasamy P., Yadav S. (2014). Changing age patterns of morbidity vis-à-vis mortality in India. J. Biosoc. Sci..

[26] Barik D., Arokiasamy P. (2016). Rising health expenditure due to non-communicable diseases in India: An Outlook. Front. Public Health.

[27] Biswas A., Singh R.K., Singh S.K. (2016). Medical and non-medical cost of hypertension and heart diseases in India. Cogent Soc. Sci..

[28] Kroll M., Phalkey R., Dutta S., Bharucha E., Butsch C., Kraas F. (2017). Urban health challenges in India: Lessons learned from a surveillance study in Pune. Erde.

[29] Paul K., Singh J. (2017). Emerging trends and patterns of self-reported morbidity in India: Evidence from three rounds of national sample survey. J. Health Popul. Nutr..

[30] Shrivastava U., Misra A., Mohan V., Unnikrishnan R., Bachani D. (2017). Obesity, diabetes and cardiovascular diseases in India: Public health challenges. Curr. Diabetes Rev..

[31] Banerjee M. (2002). Power, culture and medicine: Ayurvedic pharmaceuticals in the modern market. Contrib. Indian Sociol..

[32] Keats S., Wiggins S. (2014). Future Diets: Implications for Agriculture and Food Prices.

[33] Yadav S., Arokiasamy P. (2014). Understanding epidemiological transition in India. Glob. Health Action.

[34] Leslie C.M., Young A. (1992). Paths to Asian Medical Knowledge.

[35] Khalikova V.R. (2017). Institutionalized Alternative Medicine in North India: Plurality, Legitimacy and Nationalist Discourses. Ph.D. Thesis.

[36] Alter J.S. (2000). Gandhi’s Body: Sex, Diet, and the Politics of Nationalism.

[37] Alter J.S., Princeton N.J. (2004). Yoga in Modern India: The Body between Science and Philosophy.

[38] Khalikova V.R. (2017). The Ayurveda of Baba Ramdev: Biomoral consumerism, national duty and the biopolitics of ‘homegrown’ medicine in India. South Asia-J. South Asian Stud..

[39] Chitralekha, Pangit N., Nainwal S. (2004). Destination Wellness: Yoga, Meditation and Ayurveda in Uttaranchal.

[40] India, Ministry of Tourism (2006). Yoga, Ayurveda and Naturopathy.

[41] Langford J. (2002). Fluent Bodies: Ayurvedic Remedies for Postcolonial Imbalance.

[42] Berger R. (2013). Ayurveda Made Modern: Political Histories of Indigenous Medicine in North India, 1900–1955.

[43] Alter J.S., Baier K., Maas P.A. (2018). Nature cure and “perfect” health: The purity of the fluid body in an impure world. Yoga in Transformation: Historical and Contemporary Perspectives on a Global Phenomenon. Wiener Forum für Theologie und Religionswissenschaft.

[44] Singleton M. (2010). Yoga Body: The Origins of Modern Posture Practice.

[45] Jain A.R. (2015). Selling Yoga: From Counterculture to Pop Culture.

[46] Singleton M., Byrne J. (2008). Yoga in the Modern World: Contemporary Perspectives.

[47] Bairy S., Kumar A.M.V., Raju M., Achanta S., Naik B., Tripathy J.P., Zachariah R. (2016). Is adjunctive naturopathy associated with improved glycaemic control and a reduction in need for medications among Type 2 Diabetes patients? A prospective cohort study from India. BMC Complement. Altern. Med..

[48] Government of India (GOI) (2008). Memorandum Dated 01/01/2008: Empanelment of AYUSH Hospitals/Centers under CGHS and CS (MA) Rules for Ayurveda, Unani and Yoga and Naturopathy Treatments/Procedures and Fixation of Package Rates.

[49] Bhikha R., Glynn J. (2017). The Role of *tibb* in integrative medicine for diseases of lifestyle. Bangladesh J. Med. Sci..

[50] Josyula K.L., Sheikh K., Nambiar D., Narayan V.V., Sathyanarayana T.N., Porter J.D.H. (2016). “Getting the water-carrier to light the lamps”: Discrepant role perceptions of traditional, complementary, and alternative medical practitioners in government health facilities in India. Soc. Sci. Med..

[51] Rudra S., Kalra A., Kumar A., Joe W. (2017). Utilization of alternative systems of medicine as health care services in India: Evidence on AYUSH care from NSS 2014. PLoS ONE.

[52] Samal J., Dehury R.K. (2016). An evaluation on medical education, research and development of AYUSH systems of medicine through Five Year Plans of India. J. Clin. Diagn. Res..

[53] Wilson D., Keith G., Harpal B., Ram S., Meester Fabien D., Agnieszka W., Toru T. (2017). Therapy through social medicine: Cultivating connections and inspiring solutions for healthy living. Aims Med. Sci..

